# Chronic kidney disease (CKD) and associated risk in rural South Africa: a population-based cohort study

**DOI:** 10.12688/wellcomeopenres.18016.2

**Published:** 2022-11-03

**Authors:** June Fabian, Mwawi Gondwe, Nokthula Mayindi, Shingirai Chipungu, Bongekile Khoza, Petra Gaylard, Alisha N Wade, F. Xavier Gómez-Olivé, Laurie A Tomlinson, Michele Ramsay, Stephen Tollman, Cheryl Winkler, Jaya A George, Saraladevi Naicker

**Affiliations:** 1 Medical Research Council/Wits University Rural Public Health and Health Transitions Research Unit (Agincourt), School of Public Health, Faculty of Health Sciences, University of the Witwatersrand, Johannesburg, Gauteng, 2193, South Africa; 2 Wits Donald Gordon Medical Centre, School of Clinical Medicine, Faculty of Health Sciences, University of Witwatersrand, Johannesburg, Gauteng, 2193, South Africa; 3 Data Management and Statistical Analysis, (DMSA), Johannesburg, Gauteng, 2193, South Africa; 4 Department of non-communicable disease epidemiology, Faculty of Epidemiology and Population Health, London School of Hygiene and Tropical Medicine, London, WC1E 7HT, UK; 5 Sydney Brenner Institute for Molecular Bioscience, Faculty of Health Sciences, University of the Witwatersrand, Johannesburg, Gauteng, 2193, South Africa; 6 International Network for the Demographic Evaluation of Populations and their Health, (INDEPTH), Accra, Ghana; 7 Molecular Genetic Epidemiology Section, Basic Research Laboratory, Frederick National Laboratory for Cancer Research, NCI, Frederick, MD 21701, USA; 8 Department of Chemical Pathology, Faculty of Health Sciences, University of Witwatersrand, Johannesburg, Gauteng, 2193, South Africa; 9 Department of Chemical Pathology, National Health Laboratory Service, Johannesburg, Gauteng, 2193, South Africa; 10 Department of Internal Medicine, School of Clinical Medicine, Faculty of Health Sciences, University of Witwatersrand, Johannesburg, Gauteng, 2193, South Africa

**Keywords:** chronic kidney disease, Africa, South Africa, hypertension, HIV infection, diabetes, apolipoprotein L1

## Abstract

**Background:** In Africa, true prevalence of chronic kidney disease (CKD) is unknown, and associated clinical and genetic risk factors remain understudied. This population-based cohort study aimed to investigate CKD prevalence and associated risk factors in rural South Africa.

**Methods:** A total 2021 adults aged 20-79 years were recruited between 2017-2018 from the Agincourt Health and Socio-Demographic Surveillance System in Bushbuckridge, Mpumalanga, South Africa. The following were collected: sociodemographic, anthropometric, and clinical data; venous blood samples for creatinine, hepatitis B serology; DNA extraction; spot urine samples for dipstick testing and urine albumin: creatinine ratio (UACR) measurement. Point-of-care screening determined prevalent HIV infection, diabetes, and hypercholesterolemia. DNA was used to test for apolipoprotein L1 ( *APOL1*) kidney risk variants. Kidney Disease Improving Global Outcomes (KDIGO) criteria were used to diagnose CKD as low eGFR (<60mL/min/1.73m ^2^) and /or albuminuria (UACR ≥ 3.0mg/mmol) confirmed with follow up screening after at least three months. eGFR was calculated using the CKD-EPI _(creatinine)_ equation 2009 with no ethnicity adjustment. Multivariable logistic regression was used to model CKD risk.

**Results:** The WHO age-adjusted population prevalence of CKD was 6.7% (95% CI 5.4 - 7.9), mostly from persistent albuminuria. In the fully adjusted model, *APOL1 *high-risk genotypes (OR 2.1; 95% CI 1.3 - 3.4); HIV infection (OR 1.8; 1.1 - 2.8); hypertension (OR 2.8; 95% CI 1.8 - 4.3), and diabetes (OR 4.1; 95% CI 2.0 - 8.4) were risk factors. There was no association with age, sex, level of education, obesity, hypercholesterolemia, or hepatitis B infection. Sensitivity analyses showed that CKD risk factor associations were driven by persistent albuminuria, and not low eGFR. One third of those with CKD did not have any of these risk factors.

**Conclusions:** **In rural South Africa, CKD is prevalent, dominated by persistent albuminuria, and associated with *APOL1 *high-risk genotypes, hypertension, diabetes, and HIV infection.

## Introduction

Infectious and non-communicable disease comprise substantial risk for chronic kidney disease (CKD) in Africa, but its true prevalence remains unknown. Methodological differences in sampling frames and criteria used to diagnose CKD, and limited understanding of the best measures to assess kidney function in African populations make prevalence data difficult to interpret. Recent large epidemiological studies have highlighted regional differences in CKD prevalence - which was lower in West Africa (Ghana and Burkina Faso) compared to East (Kenya) and South Africa, and higher in eastern compared to southern Uganda ^1, 2^.

Risk factors associated with kidney disease are understudied. In many African studies traditional risk factors associated with CKD include hypertension, diabetes, HIV infection, older age, and female sex ^1, 3^. Studies from Tanzania, Malawi, Uganda and Kenya, suggest non-traditional risk factors are an important contributor to CKD risk ^2, 4, 5^. These include endemic and other infectious diseases, such as undiagnosed genitourinary tuberculosis (TB), schistosomiasis, and viruses other than human immunodeficiency virus (HIV) which can manifest as nitrite-negative leukocyturia and/or hematuria, or tubulointerstitial injury related to occupational or environmental toxin exposure ^3, 5^.

Compared to other US populations groups, African Americans have a three-to-four times higher risk of kidney failure associated with recessive inheritance of apolipoprotein L1 ( *APOL1*) kidney risk variants (KRV) ^6^. *APOL1* KRV comprise two missense single nucleotide polymorphisms (SNPs) defining the G1 allele and a six base pair-deletion defining the G2 allele. G1 and G2 alleles originated in West Africa with recent positive selection from protection against trypanosomal African sleeping sickness. *APOL1* KRV frequencies vary widely in Africa: Nigeria's Igbo and Yoruba people have the highest frequencies (40%), with lower frequencies in South Africa (18%), and near-absence in East Africa ^7^.

The role of *APOL1* KRV in the pathogenesis of CKD in African populations is unclear. *APOL1* KRV have been associated with hypertension-attributed and non-diabetic CKD in Democratic Republic of Congo and Nigeria, persistent albuminuria despite well-controlled HIV disease in Nigeria, and HIV-associated nephropathy, systolic hypertension and low eGFR in South Africa ^2, 8– 11^. One familial study from South African failed to demonstrate an association between *APOL1* KRV and hypertension-attributed CKD compared to unaffected family members ^12^. Recently, a large population-based study showed an association between *APOL1* KRV and albuminuria (but not eGFR), and this association was attenuated when compared to African American populations ^13^.

The aim of this study was to determine the prevalence of CKD and identify associated clinical and genetic risk factors in a rural South African population. We hypothesized that CKD prevalence would be high and associated with *APOL1* KRV, infectious and non-communicable disease.

## Methods

### Study setting and sampling strategy

This longitudinal cohort study was conducted from November 2017 to September 2018 in the Medical Research Council (MRC)/Wits Rural Public Health and Health Transitions Research Unit (otherwise referred to as "Agincourt") in Bushbuckridge, a rural subdistrict of the Mpumalanga province in north-eastern South Africa ^14^. Agincourt is a health and socio-demographic surveillance system (HDSS) site that includes approximately 115,000 people. A minimum sample size of 1800 was required to provide at least 80% power to determine CKD prevalence of at least 5%, provided the true prevalence was equal to or more than 6.5%. Proportional allocation of Black African adults aged 20 to 79 years ensured a representative sample based on the most recent annual population census. Sample size was increased proportionately to 2759 individuals to accommodate a 25% non-participation rate. 

### Participant recruitment and study procedures

Ethics approval was obtained from the Medical Human Research Ethics Committee, University of the Witwatersrand (certificate number M170583). Trained fieldworkers and nurses performed home visits in 31 villages comprising the Agincourt HDSS. Written informed consent was obtained in the participant's first language (primarily Xitsonga). Participants with abnormal tests were referred to their local primary health care clinic for confirmatory testing and further management. Weight (kg) was measured using a digital scale and height was measured with a portable stadiometer (Seca, Germany). Height and weight were used to calculate body mass index (BMI) ((kg)/(m) ^2^. Blood pressure (BP) was measured using automated upper arm devices (Omron M6W, Intellisense BP785 large cuff, Japan). Participants were asked to sit comfortably with legs uncrossed for five minutes before taking readings. Three measurements were taken using an appropriate-sized cuff on the left arm at two-minute intervals. Of three BP readings, the first was discarded, and a mean of the second and third readings used for analyses. Nurses performed capillary point of care (POC) random cholesterol and random glucose testing (Cardiochek PA analyzer, PTS Panels test strips, PTS Diagnostics, USA). If a participant knew their HIV status as positive, this was recorded. If HIV status was unknown or participants previously tested negative, nurses offered voluntary POC screening and testing (Alere HIV Combo, Abbott, USA) according to South African Department of Health guidelines ^15^. A positive test result was confirmed with a second test (Uni-Gold Recombigen HIV-1/2, Trinity BioTech, USA). Nurses collected blood samples and a freshly voided urine sample for laboratory testing. A spot urine pregnancy test was performed for premenopausal women (Abon One Step Pregnancy Test, Pharmaland, UAE). Samples were stored in isothermal bags (2 – 6 °C) with temperature monitoring (Easylog, Lascar Electronics, UK). After completing fieldwork, samples were delivered to the Agincourt Research laboratory for processing and storage at -80°C according to standard operation procedures.

### Laboratory procedures

A 20µL aliquot of DNA was shipped to the Frederick National Laboratory at the National Cancer Institute, USA, for *APOL1* genotyping ^16^. DNA was genotyped using TaqMan assays (ThermoFisher Scientific, USA). *APOL1* G1 KRV comprised a missense G nucleotide at rs73885319 (G1g) and either a T or G nucleotide at rs60910145; presence of only the G1g (p.342Gly) variant was sufficient to define the G1 KRV ^17^. The *APOL1* G2 KRV consists of a six-base-pair in-frame deletion, rs717185313. The number of *APOL1* KRV (G1 or G2) carried by each participant was coded as 0 for the G0/G0 genotype, 1 for the G0/G1 or G0/G2 genotype, or 2 for the G1/G1, G1/G2, or G2G2 genotypes. *APOL1* genotypes were further coded as "high-risk (HR)" if the participant carried any combination of 2 risk alleles or "low-risk (LR)" if the participant had 0 or 1 risk allele. This classification was used for statistical analyses ^16^. All remaining specimens were shipped at -80°C to the Central Laboratory Services (CLS) in Johannesburg, South Africa. Serum and urine creatinine was measured by an isotope-dilution mass spectrometry traceable modified Jaffe method, urine albumin by a colorimetric method (Cobas 6000/c501 analyzer), urine albumin:creatinine ratios (UACR) were calculated and reported (mg/mmol), and hepatitis B status was determined using Immulite serological assays (ARCHITECT i1000SR analyzer, Abbott USA). The CLS laboratory adhered to standard daily internal quality control procedures and complied with the requirements of the external quality control program through the College of American Pathologists.

### Study procedures

For each participant, highest level of education was received from the Agincourt HDSS. Body mass index (BMI) was used to classify participants as underweight (< 18.5); normal (18.5 – 24.9); overweight (25.0 – 29.9); or obese (≥ 30.0) ^18^. Participant blood pressure was classified according to the 7 ^th^ Report of the Joint National Committee on Prevention, Detection, Evaluation, and Treatment of High Blood Pressure as normotensive: systolic blood pressure (SBP) <120mmHg and diastolic blood pressure (DBP) <80mmHg; prehypertensive: SBP ≥120mmHg and <140mmHg or DBP ≥80mmHg and <90mmHg; and hypertensive: SBP ≥ 140mmHg or DBP ≥ 90mmHg ^19^. Diabetes was defined as a non-fasting glucose ≥ 11.1mmol/L; and hypercholesterolaemia as a non-fasting total cholesterol > 5.0mmol/L ^20, 21^.

### Chronic kidney disease prevalence

Kidney Disease Improving Global Outcomes (KDIGO) criteria were used to diagnose CKD ^22^. eGFR was calculated using the CKD-EPI _(creatinine)_ equation 2009 without adjusting for African American ethnicity as race-based coefficients have been shown to overestimate GFR in this rural South African population ^23^. Albuminuria was quantified with spot UACR. Participants with low eGFR (<60ml/min/1.73m ^2^), and/or albuminuria (UACR ≥3.0mg/mmol) were followed up with repeated measures after a minimum of three months. CKD was defined as low eGFR, or albuminuria, or a combination (low eGFR and/or albuminuria) provided these measures were confirmed on repeat testing, and this definition was used for all analyses. 

### Statistical analysis

Continuous variables were represented as mean (standard deviation [SD]) if normally distributed and median (interquartile range) if non-normally distributed. Categorical variables were expressed as frequencies (percentage). Study variables were compared between sexes using the chi-squared test (Fisher’s exact test was used for 2×2 tables). To identify factors associated with CKD, logistic regression analysis was used to estimate odds ratios (OR), with corresponding 95% confidence intervals (CIs). Hierarchical models based on existing knowledge of known CKD risk factors were developed with all models, age- and sex-adjusted. Model 1 incorporated level of education and BMI. Model 2 added *APOL1* genotype status, and Model 3 added comorbid infectious and non-communicable conditions: hepatitis B, HIV, hypertension, diabetes, and hypercholesterolaemia. Nested models were compared using the likelihood ratio test. Because CKD was a composite variable (low eGFR and/or albuminuria), sensitivity analyses compared whether there were differences in association between risk factors and (i) low GFR alone, or (ii) albuminuria alone. Missing data were reported in figures and tables. CKD population prevalence was age-standardized using the revised World Health Organization (WHO) World Standard Population Distribution for ages 20–79 (direct method) ^24, 25^. Statistical analyses were performed using SAS (Stata Corp, Texas, USA) and can be performed in R (R Core Team, 2014) ^26^.

## Results

**Figure 1.**
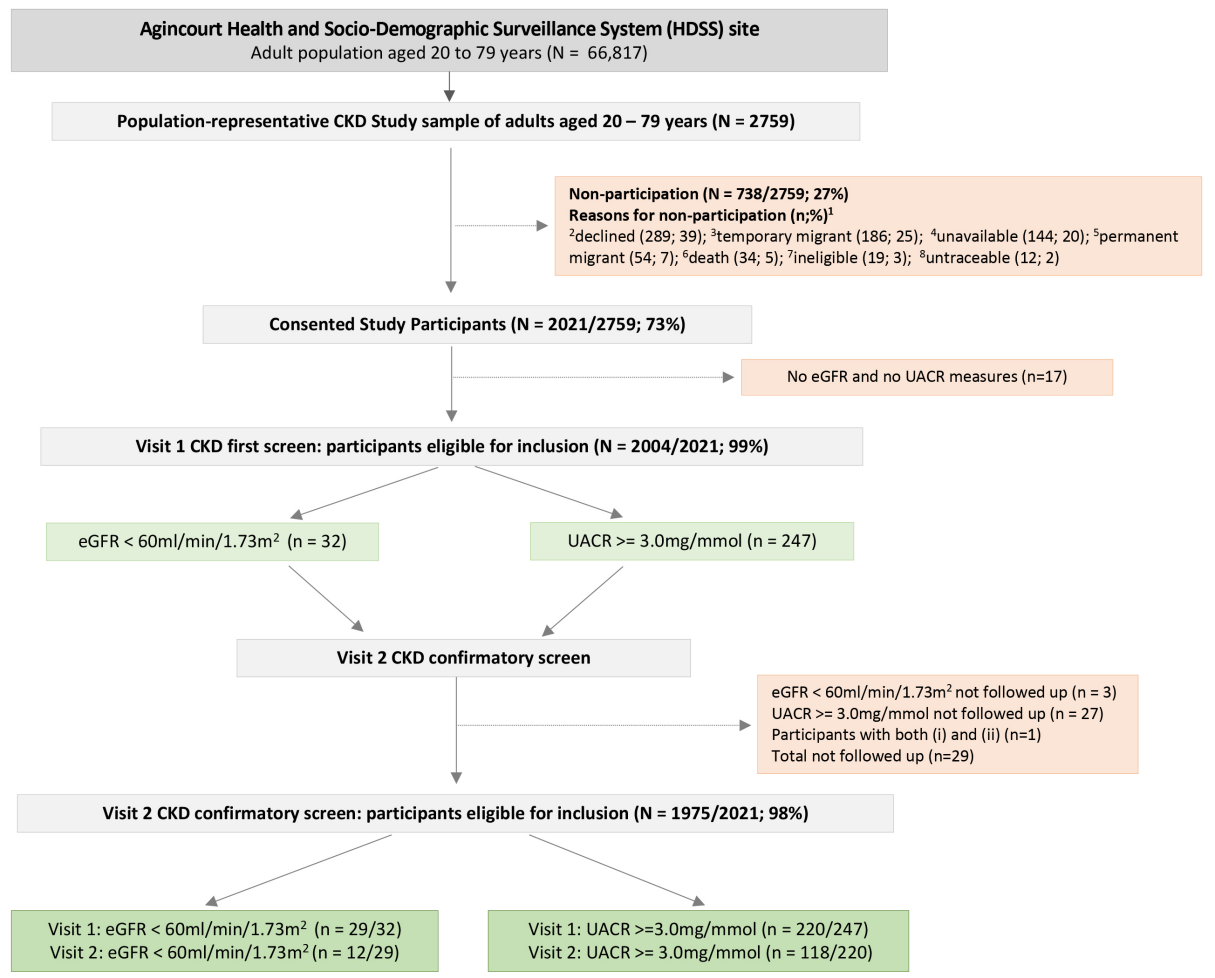
Flow diagram depicting study sample selection, participant recruitment, and CKD screening strategy.

**Figure 2.**
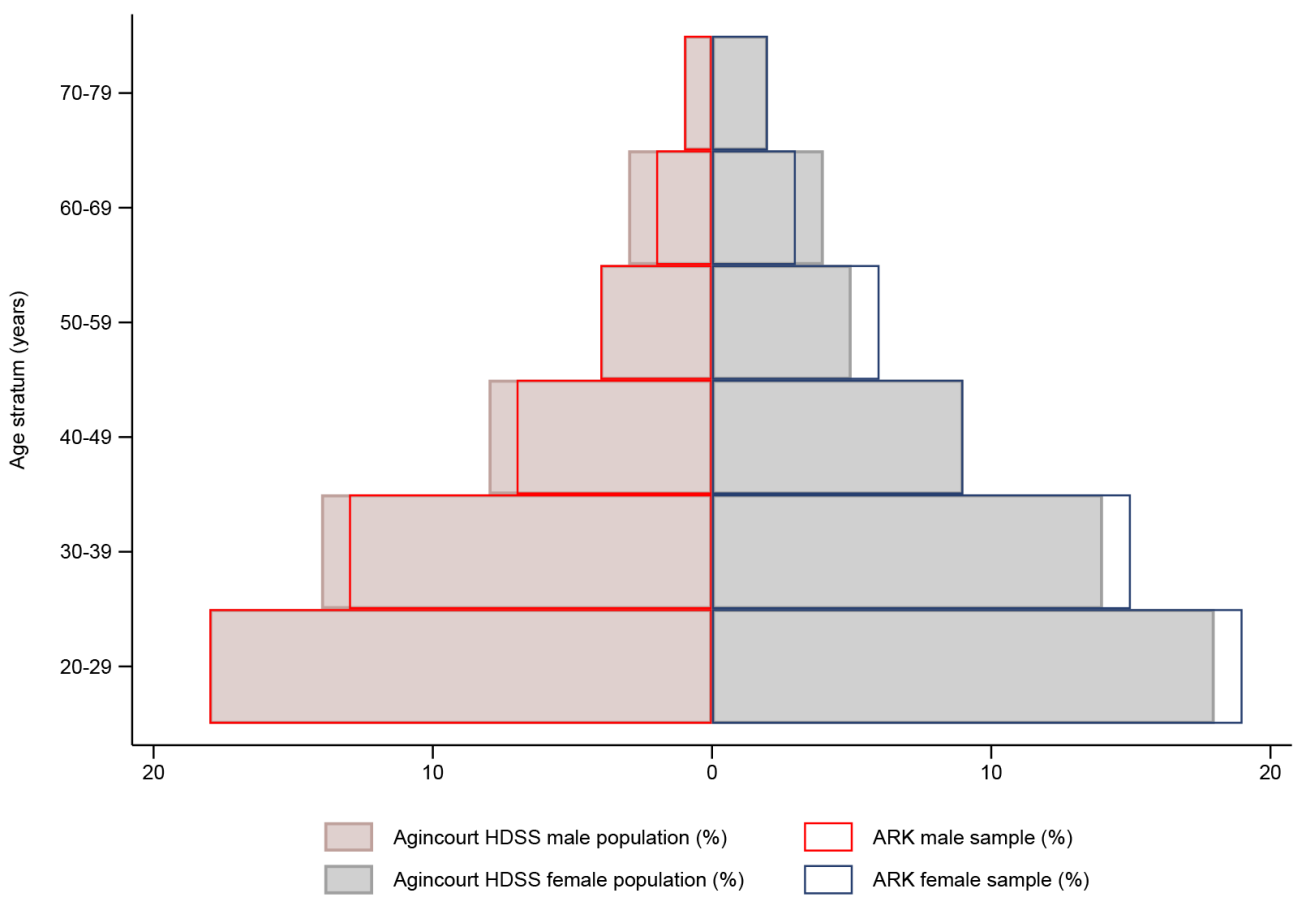
Comparison of the study sample with Agincourt HDSS population stratified by age and sex.

The flow diagram in Figure 1 details sample selection, reasons for non-participation, and CKD screening procedures. Overall, 2021/2759 adults consented (73% participation rate), with the final study sample representative of the Agincourt HDSS population ( Figure 2). Participant socio-demographic and clinical characteristics overall, and stratified by sex, are summarized in Table 1. For participants with complete data for eGFR and UACR (n=2004): 32 had low eGFR at first screening, and of these, 12/29 (41%) were confirmed with low eGFR at follow-up; 247 had albuminuria at first screening, and of these, 118/220 (54%) were confirmed with albuminuria at follow-up ( Figure 1). Overall, the WHO age-standardized prevalence for low eGFR was 0.9% (95% CI 0.4 - 1.4); for albuminuria was 6.2% (95% CI 5.0 - 7.4), and for CKD (low eGFR and/or albuminuria) was 6.7% (95% CI 5.4 - 7.9).

**Table 1.** Sociodemographic and clinical characteristics of the study participants.

Variable ^1^	Men, N (%) (N = 851)	Women, N (%) (N = 1170)	Overall, N (%) (N = 2021)
**Age (years)** median (IQR)	35 (27 – 47)	34 (25 – 45)	35 (27 – 48)
20 – 39 years N (%)	557 (65.5)	705 (60.3)	1262 (62.4)
40 – 59 years N (%)	227 (26.7)	329 (28.1)	556 (27.5)
60 – 79 years N (%)	67 (7.9)	136 (11.6)	203 (10.0)
**^2^Serum creatinine (µmol/L)** mean (SD)	73 (15)	56 (13)	63 (16)
**^2^estimated GFR (ml/min/1.73m ^2^)** mean (SD)	112 (17)	112 (19)	112 (18)
**^3^UACR (mg/mmol)** median (IQR)	0.3 (0.2 – 1.0)	0.5 (0.3 – 1.3)	0.4 (0.2 – 1.2)
**^4^Highest education level**			
No formal education N (%)	47 (5.6)	135 (11.7)	182 (9.1)
Less than six years completed N (%)	65 (7.7)	94 (8.1)	159 (7.9)
Six or more years completed N (%)	733 (86.8)	928 (80.2)	1661 (83.0)
**^5^BMI (kg/m ^2^)**			
Normal (BMI 18.5 - 24.99)	493 (58.0)	277 (24.9)	770 (39.2)
Underweight (BMI < 18.5)	55 (6.5)	28 (2.5)	83 (4.2)
Overweight (BMI (25.0 - 29.99)	206 (24.2)	365 (32.8)	571 (29.1)
Obese (BMI >= 30.0)	96 (11.3)	444 (39.9)	540 (27.5)
**^6^ *APOL1* Low-risk** (zero or one risk allele) N (%)	745 (88.3)	1031 (89.0)	1776 (88.7)
**^6^ *APOL1* High-risk** (two risk alleles) N (%)	99 (11.7)	127(11.0)	226 (11.3)
**^7^Blood pressure**			
Normal N (%)	212 (24.9)	543 (46.4)	755 (37.4)
Pre-hypertension N (%)	452 (53.1)	445 (38.0)	897 (44.4)
Hypertension	187 (22.0)	182 (15.6)	369 (18.3)
**^8^Diabetes** N (%)	21 (2.5)	50 (4.3)	71 (3.5)
**^9^Hypercholesterolaemia** N (%)	144 (16.9)	321 (27.4)	465 (23.0)
**^10^Hepatitis B infection** N (%)	40 (4.7)	40 (3.4)	80 (4.0)
**^11^HIV infection** N (%)	102 (12.0)	278 (23.8)	380 (18.8)
**^12^Normoalbuminuric nitrite negative leukocyturia** N (%)	124 (16.9)	354 (38.0)	478 (28.7)
**^13^Normoalbuminuric haematuria** N (%)	179 (24.4)	294 (31.6)	473 (28.4)

^1^Percentages may sum to +/- 100 from rounding; for missing data : ^2^Serum creatinine and estimated GFR from first screening: n=1169 for women; n=2020 overall; ^3^UACR: urine albumin: creatinine ratio from first screening: n=845 for men; n=1160 for women; n=2005 overall; ^4^Highest level of education: n=845 for men; n=1157 for women; n=2002 overall; ^5^Body mass index (BMI) = weight (kg)/height (m ^2^): excluded pregnant women n=53; n=850 for men; n=1114 for women; n=1964 overall; ^6^ *APOL1* risk genotypes: n=844 for men; n=1158 for women; n=2002 overall; ^7^normal: SBP < 120mmHg and DBP < 80mmHg; pre-hypertension: SBP ≥ 120mmHg and < 140mmHg or DBP ≥ 80mmHg and < 90mmHg; hypertension: SBP ≥ 140mmHg or DBP ≥ 90mmHg; women with hypertension: 90/182 (49.5%) were previously tested and informed they were hypertensive; 78/182 (42.9%) were previously tested and informed they were normotensive; 14/182 (7.7%) had not been tested; men with hypertension: 55/187 (29.4%) were previously tested and informed they were hypertensive; 93/187 (49.7%) were previously tested and informed they were normotensive; 39/187 (20.9%) had not been tested; ^8^Diabetes: non-fasting glucose >= 11.1mmol/L; ^9^Hypercholesterolaemia: non-fasting total cholesterol > 5.0mmol/L; ^10^Hepatitis B infection: n=851 for men; n=1169 for women; n=2020 overall; ^11^HIV Infection: women testing positive: 259/278 (93.2%) had prior knowledge of their status; 15/278 (5.4%) had previously tested negative; 4/278 (1.4%) had no prior testing; men testing positive: 88/102 (86.2%) had prior knowledge of their status; and 7/102 (6.9%) each, had previously tested negative or had no prior testing. ^12^Urine dipstick results from first screening; excluded pregnant women n=53; n=734 for men; n=931 for women; n=1665 overall; ^13^Urine dipstick results from first screening; excluded pregnant women n=53; n=735 for men; n=931 for women; n=1666 overall.

Results from multivariable adjusted logistic regression analyses are summarised in Table 2. In the fully adjusted model, CKD was associated with diabetes (OR 4.0; 95% CI 1.9 - 8.3), hypertension (OR 2.8; 95% CI 1.8 - 4.3), high-risk *APOL1* genotype (OR 2.1; 95% CI 1.3 - 3.4), and HIV infection (OR 1.8; 95% CI 1.1 - 2.8). CKD was not associated with age, sex, level of education, BMI, hepatitis B infection or hypercholesterolaemia. Because CKD was a composite variable, sensitivity analyses were conducted to determine whether the associations observed were driven by low eGFR or albuminuria. The number of events was too small for a sensitivity analysis restricted to eGFR <60mL/min/1.73m ^2^. Instead, a sensitivity analysis was performed using eGFR <90mL/min/1.73m ^2^ on initial screen which showed an association with advancing age, obesity, and diabetes, but not with *APOL1* high-risk genotypes, HIV infection, or hypertension ( Table 3). For albuminuria, two sensitivity analyses were performed for those with albuminuria on (i) initial screening, and (ii) follow up screening ( Table 4– Table 5). Both confirmed associations observed with the composite endpoint (CKD defined as low eGFR and/or albuminuria) were primarily driven by persistent albuminuria. 

For participants with CKD, overall, there was no identified risk factor in 32% (37/117) of participants ( Table 6), most had one risk factor, and none had more than three. Women had fewer identified risk factors than men. CKD risk factors included those identified in the multivariable regression analyses: high-risk *APOL1* genotype, hypertension, HIV infection, and diabetes. 

**Table 2.** CKD prevalence and model-adjusted odds ratios for CKD risk by socio-demographic factors, *APOL1* status, infectious and non-communicable comorbidity.

			All models age- and sex-adjusted		
Variable	Overall N = 1975 ^1^ n (%)	CKD N = 124 (6.3%) n (%)	Model 1 ^2^ Adjusted for education, BMI	Model 2 ^2^ Adjusted for education, BMI, *APOL1* genotype	Model 3 ^2^ Adjusted for education, BMI, *APOL1* genotype, hepatitis B, HIV status, hypertension, diabetes, hypercholesterolemia
**Age (years)**					
20 – 39	1234 (62.5)	70 (5.7)	1.00 *reference*	1.00 *reference*	1.00 *reference*
40 – 59	546 (27.6)	34 (6.2)	1.07 (0.67 – 1.68)	1.08 (0.68 – 1.72)	0.70 (0.43 – 1.15)
60 – 79	195 (9.9)	20 (10.3)	1.52 (0.76 – 3.07)	1.55 (0.77 – 3.13)	0.82 (0.38 – 1.76)
**Sex**					
Male	829 (42.0)	55 (6.6)	1.00 *reference*	1.00 *reference*	1.00 *reference*
Female	1146 (58.0)	69 (6.0)	0.85 (0.57 – 1.26)	0.85 (0.57 – 1.27)	0.88 (0.58 – 1.34)
**Education**					
No education	179 (9.2)	14 (7.8)	1.00 *reference*	1.00 *reference*	1.00 *reference*
Less than six years	155 (7.9)	15 (9.7)	1.42 (0.65 – 3.13)	1.38 (0.63 – 3.04)	1.24 (0.55 – 2.80)
Six or more years	1622 (82.9)	93 (5.7)	0.93 (0.44 – 1.93)	0.91 (0.43 – 1.89)	0.86 (0.41 – 1.81)
**^3^BMI (kg/m ^2^)**					
Non-obese	1388 (72.4)	89 (6.4)	1.00 *reference*	1.00 *reference*	1.00 *reference*
Obese	530 (27.6)	31 (5.8)	0.89 (0.56– 1.42)	0.87 (0.55 – 1.39)	0.67 (0.41 – 1.09)
***APOL1* genotype**					
Low-risk	1737 (88.7)	99 (5.7)		1.00 *reference*	1.00 *reference*
High-risk	221 (11.3)	24 (10.9)		**2.16 (1.34 – 3.48)**	**2.10 (1.29 – 3.42)**
**Hepatitis B status**					
negative	1897 (96.1)	118 (6.2)			1.00 *reference*
positive	78 (3.9)	6 (7.7)			0.93 (0.33 – 2.63)
**HIV status**					
negative/unknown	1602 (81.1)	94 (5.9)			1.00 *reference*
positive	373 (18.9)	30 (8.0)			**1.78 (1.12 – 2.83)**
**Blood pressure**					
No hypertension	1617 (81.9)	80 (4.9)			1.00 *reference*
hypertension	358 (18.1)	44 (12.3)			**2.76 (1.78 – 4.27)**
**Diabetes**					
absent	1909 (96.7)	110 (5.8)			1.00 *reference*
present	66 (3.3)	14 (21.2)			**4.00 (1.93 – 8.29)**
**Hypercholesterolaemia**					
absent	1524 (77.2)	85 (5.6)			1.00 *reference*
present	451 (22.8)	39 (8.6)			1.37 (0.87 – 2.14)

Column percentages may sum to +/-100 due to rounding; odds ratios presented with 95% confidence intervals; categories presented as frequency (%); ^1^N = 1975: total number eligible for inclusion after CKD screening and follow up; ^2^N = 1885: total number with complete data for variables included in regression models; ^3^BMI: body mass index: non-obese <30.0; obese BMI >= 30.0; Bold text indicates 5% level of significance (p-value <0.05).

**Table 3.** eGFR <90mL/min/1.73m ^2^ (on initial screen) and model-adjusted odds ratios by socio-demographic factors, *APOL1* status, infectious and non-communicable comorbidity.

			All models age- and sex-adjusted		
Variable ^1^	Overall N = 1975 n (%)	eGFR <90 ^2^ N = 220 n (%)	Model 1 Adjusted for education, BMI	Model 2 Adjusted for education, BMI, *APOL1* genotype	Model 3 Adjusted for education, BMI, *APOL1* genotype, hepatitis B, HIV status, hypertension, diabetes, hypercholesterolemia
**Age (years)**					
20 – 39	1234 (62.5)	43 (3.5)	1.00 *reference*	1.00 *reference*	1.00 *reference*
40 – 59	546 (27.6)	81 (14.8)	**4.60 (3.08 – 6.86)**	**4.59 (3.07 – 6.85)**	**4.13 (2.73 – 6.26)**
60 – 79	195 (9.9)	96 (49.2)	**26.1 (15.5 – 43.7)**	**26.0 (15.5 – 43.7)**	**22.1 (12.9 – 37.9)**
**Sex**					
Male	829 (42.0)	90 (10.9)	1.00 *reference*	1.00 *reference*	1.00 *reference*
Female	1146 (58.0)	130 (11.3)	0.74 (0.52 – 1.04)	0.75 (0.52 – 1.04)	0.75 (0.53 – 1.07)
**Education**					
No education	179 (9.2)	53 (32.4)	1.00 *reference*	1.00 *reference*	1.00 *reference*
Less than six years	155 (7.9)	34 (21.9)	0.83 (0.48 – 1.43)	0.83 (0.48 – 1.44)	0.75 (0.53 – 1.07)
Six or more years	1622 (82.9)	127 (7.8)	0.99 (0.61 – 1.61)	1.00 (0.61 – 1.62)	0.82 (0.47 – 1.42)
**^3^BMI (kg/m ^2^)**					
Non-obese	1388 (72.4)	133 (9.6)	1.00 *reference*	1.00 *reference*	1.00 *reference*
Obese	530 (27.6)	86 (16.2)	**1.61 (1.13 – 2.28)**	**1.61 (1.13 – 2.28)**	**1.46 (1.02 – 2.49)**
***APOL1* genotype**					
Low-risk	1737 (88.7)	197 (11.3)		1.00 *reference*	1.00 *reference*
High-risk	221 (11.3)	22 (10.0)		0.93 (0.55 – 1.55)	0.93 (0.56 – 1.57)
**Hepatitis B status**					
negative	1897 (96.1)	207 (10.9)			1.00 *reference*
positive	78 (3.9)	13(16.7)			1.33 (0.65 – 2.71)
**HIV status**					
negative/unknown	1602 (81.1)	177 (11.0)			1.00 *reference*
positive	373 (18.9)	43 (11.5)			1.07 (0.71 – 1.61)
**Blood pressure**					
No hypertension	1617 (81.9)	150 (9.3)			1.00 *reference*
hypertension	358 (18.1)	70 (19.6)			1.18 (0.82 – 1.71)
**Diabetes**					
absent	1909 (96.7)	192 (10.1)			1.00 *reference*
present	66 (3.3)	28 (42.4)			**2.06 (1.13 – 3.75)**
**Hypercholesterolaemia**					
absent	1524 (77.2)	137 (9.0)			1.00 *reference*
present	451 (22.8)	83 (18.4)			1.09 (0.77 – 1.56)

^1^Column percentages may sum to +/-100 due to rounding; odds ratios presented with 95% confidence intervals; categories presented as frequency (%); ^2^eGFR<90: estimated GFR less than 90ml/min/1.73m ^2^; ^3^BMI: body mass index: non-obese <30.0; obese BMI >= 30.0; Bold text indicates 5% level of significance (p-value <0.05)

**Table 4.** Albuminuria (on initial screen) and model-adjusted odds ratios by socio-demographic factors, *APOL1* status, infectious and non-communicable comorbidity.

			All models age- and sex-adjusted		
Variable ^1^	Overall N = 1975 n (%)	Albuminuria N= 220 n (%)	Model 1 Adjusted for education, BMI	Model 2 Adjusted for education, BMI, *APOL1* genotype	Model 3 Adjusted for education, BMI, *APOL1* genotype, hepatitis B, HIV status, hypertension, diabetes, hypercholesterolemia
**Age (years)**					
20 – 39	1234 (62.5)	122 (9.9)	1.00 *reference*	1.00 *reference*	1.00 *reference*
40 – 59	546 (27.6)	70 (12.8)	1.34 (0.95 – 1.89)	1.36 (0.96 - 1.91)	1.00 (0.70 - 1.45)
60 – 79	195 (9.9)	28 (14.4)	1.33 (0.76 – 2.35)	1.34 (0.76 – 2.37)	0.81 (0.44 - 1.50)
**Sex**					
Male	829 (42.0)	84 (10.1)	1.00 *reference*	1.00 *reference*	1.00 *reference*
Female	1146 (58.0)	136 (11.9)	1.08 (0.79 – 1.48)	1.08 (0.79 - 1.48)	1.12 (0.81 - 1.55)
**Education**					
No education	179 (9.2)	22 (12.3)	1.00 *reference*	1.00 *reference*	1.00 *reference*
Less than six years	155 (7.9)	25 (16.1)	1.53 (0.81 – 2.89)	1.50 (0.80 – 2.83)	1.47 (0.76 – 2.77)
Six or more years	1622 (82.9)	171 (10.5)	1.06 (0.60 – 1.87)	1.04 (0.59 – 1.85)	1.00 (0.57 – 1.84)
**^2^BMI (kg/m ^2^)**					
Non-obese	1388 (72.4)	146 (10.5)	1.00 *reference*	1.00 *reference*	1.00 *reference*
Obese	530 (27.6)	64 (12.1)	1.08 (0.77 – 1.52)	1.07 (0.76 – 1.50)	0.90 (0.63 - 1.28)
***APOL1* genotype**					
Low-risk	1737 (88.7)	186 (10.7)		1.00 *reference*	1.00 *reference*
High-risk	221 (11.3)	33 (14.9)		**1.57 (1.05 – 2.36)**	**1.54 (1.02 – 2.33)**
**Hepatitis B status**					
negative	1897 (96.1)	209 (11.0)			1.00 *reference*
positive	78 (3.9)	11 (14.1			1.03 (0.48 - 2.21)
**HIV status**					
negative/unknown	1602 (81.1)	170 (10.6)			1.00 *reference*
positive	373 (18.9)	50 (13.4)			1.43 (0.99 - 2.06)
**Blood pressure**					
No hypertension	1617 (81.9)	155 (9.6)			1.00 *reference*
hypertension	358 (18.1)	65 (18.2)			**1.94 (1.36 – 2.76)**
**Diabetes**					
absent	1909 (96.7)	198 (10.4)			1.00 *reference*
present	66 (3.3)	22 (33.3)			**3.79 (2.08 – 6.88)**
**Hypercholesterolaemia**					
absent	1524 (77.2)	154 (10.1)			1.00 *reference*
present	451 (22.8)	66 (14.6)			1.26 (0.89 – 1.79)

^1^Column percentages may sum to +/-100 due to rounding; odds ratios presented with 95% confidence intervals; categories presented as frequency (%); ^2^BMI: body mass index: non-obese <30.0; obese BMI >= 30.0; Bold text indicates 5% level of significance (p-value <0.05).

**Table 5.** Albuminuria (confirmed with follow up) and model-adjusted odds ratios by socio-demographic factors, *APOL1* status, infectious and non-communicable comorbidity.

			All models age- and sex-adjusted		
Variable ^1^	Overall N = 1975 n (%)	Albuminuria N = 118 n (%)	Model 1 Adjusted for education, BMI	Model 2 Adjusted for education, BMI, *APOL1* genotype	Model 3 Adjusted for education, BMI, *APOL1* genotype, hepatitis B, HIV status, hypertension, diabetes, hypercholesterolemia
**Age (years)**					
20 – 39	1234 (62.5)	69 (5.6)	1.00 *reference*	1.00 *reference*	1.00 *reference*
40 – 59	546 (27.6)	33 (6.0)	1.02 (0.64 – 1.63)	1.05 (0.65 - 1.68)	0.69 (0.42 – 1.15)
60 – 79	195 (9.9)	16 (8.2)	1.11 (0.52 – 2.36)	1.13 (0.53 – 2.40)	0.61 (0.27 – 1.48)
**Sex**					
Male	829 (42.0)	53 (6.4)	1.00 *reference*	1.00 *reference*	1.00 *reference*
Female	1146 (58.0)	65 (5.7)	0.82 (0.54 – 1.23)	0.82 (0.55 - 1.24)	0.85 (0.55 – 1.30)
**Education**					
No education	179 (9.2)	13 (7.3)	1.00 *reference*	1.00 *reference*	1.00 *reference*
Less than six years	155 (7.9)	13 (8.4)	1.25 (0.55 – 2.87)	1.21 (0.53 – 2.78)	1.08 (0.46 – 2.54)
Six or more years	1622 (82.9)	90 (5.5)	0.81 (0.38 – 1.73)	0.79 (0.37 – 1.70)	0.76 (0.35 – 1.63)
**^2^BMI (kg/m ^2^)**					
Non-obese	1388 (72.4)	84 (6.1)	1.00 *reference*	1.00 *reference*	1.00 *reference*
Obese	530 (27.6)	30 (5.7)	0.95 (0.59 - 1.51)	0.92 (0.58 – 1.48)	0.72 (0.44 - 1.18)
***APOL1* genotype**					
Low-risk	1737 (88.7)	93 (5.4)		1.00 *reference*	1.00 *reference*
High-risk	221 (11.3)	24 (10.9)		**2.31 (1.43 – 3.72)**	**2.23 (1.37 – 3.64)**
**Hepatitis B status**					
negative	1897 (96.1)	112 (5.9)			1.00 *reference*
positive	78 (3.9)	6 (7.7)			0.97 (0.34 - 2.77)
**HIV status**					
negative/unknown	1602 (81.1)	90 (5.6)			1.00 *reference*
positive	373 (18.9)	28 (7.5)			**1.70 (1.06 - 2.73)**
**Blood pressure**					
No hypertension	1617 (81.9)	78 (4.8)			1.00 *reference*
hypertension	358 (18.1)	40 (11.2)			**2.56 (1.63 – 4.00)**
**Diabetes**					
absent	1909 (96.7)	106 (5.6)			1.00 *reference*
present	66 (3.3)	12 (18.2)			**3.54 (1.64 – 7.60)**
**Hypercholesterolaemia**					
absent	1524 (77.2)	81 (5.3)			1.00 *reference*
present	451 (22.8)	37 (8.2)			1.45 (0.92 – 2.30)

^1^Column percentages may sum to +/-100 due to rounding; odds ratios presented with 95% confidence intervals; categories presented as frequency (%); ^2^BMI: body mass index: non-obese <30.0; obese BMI >= 30.0; Bold text indicates 5% level of significance (p-value <0.05).

**Table 6.** Distribution of risk factors in those with and without CKD overall, and by sex.

Number of CKD risk factors ^1^	Overall N (%)	No CKD N(%)	CKD N (%)
**Overall**	**N = 1885**	**N = 1768**	**N = 117**
**0**	1059 (56.2)	1022 (57.8)	37 (31.6)
**1**	682 (36.2)	628 (35.5)	54 (46.2)
**2**	131 (6.9)	109 (6.2)	22 (18.8)
**3**	13 (0.7)	9 (0.5)	4 (3.4)
**Men**	**N = 815**	**N = 760**	**N = 55**
**0**	482 (59.1)	467 (61.4)	15 (27.3)
**1**	280 (34.4)	251 (33.0)	29 (52.7)
**2**	47 (5.8)	38 (5.0)	9 (16.4)
**3**	6 (0.7)	4 (0.5)	2 (3.6)
**Women**	**N = 1070**	**N = 1008**	**N = 62**
**0**	577 (53.9)	555 (55.1)	22 (35.5)
**1**	402 (37.6)	377 (37.4)	25 (40.3)
**2**	84 (7.9)	71 (7.0)	13 (21.0)
**3**	7 (0.7)	5 (0.5)	2 (3.2)

*^1^CKD risk factors: from multivariable regression analysis we identified as APOL1 high-risk genotype; HIV infection, hypertension, and diabetes*.

## Discussion

This rigorously conducted study determined CKD prevalence in a rural South African population using recommended KDIGO criteria for eGFR and albuminuria with confirmatory testing. Far more than low eGFR, persistent albuminuria was the dominant kidney function abnormality and the primary driver of observed associations with *APOL1* high-risk genotypes, HIV infection, hypertension, and diabetes. There were no significant associations with age, sex, level of education, obesity, hepatitis B infection, or hypercholesterolemia.

There are several strengths to the study including the rural population-based sampling frame, combined evaluation of eGFR and albuminuria, and confirmation with follow-up testing which reduced the risk of over-reporting prevalent kidney disease. The strong contribution of persistent albuminuria to CKD prevalence is relevant, as many large epidemiological studies rely solely on the estimation of GFR. Limitations include evaluation of few non-traditional risk factors for CKD, and low power for evaluating risk in those with eGFR <60mL/min/1.73m ^2^. Participants with hypertension, HIV (not on treatment), anaemia, hypercholesterolaemia, and hyperglycaemia were referred to their local care facility for further investigation and/or treatment. In so doing, those with concomitant low eGFR and / or albuminuria at baseline might have received treatment that affected subsequent kidney function testing. The relatively small proportion of participants with low eGFR might be explained by the absence of appropriate care for those with severe kidney disease, thus creating a survival bias, or potential to overestimate creatinine-based GFR with the CKD-EPI _(creatinine) _ equation with consequent underdiagnosis of CKD ^27^.

The association of *APOL1* high-risk genotypes with persistent albuminuria is consistent with population-based studies in continental African and African American populations ^13, 28, 29^. While the population frequencies of *APOL1* high-risk genotypes approximated those reported in African Americans (~10–15%) and the association with persistent albuminuria similar, our study did not show any association with low eGFR ^7^. This might relate to limited analytic power because so few had low eGFR, but it is worth noting that similar findings have been described in a population-based study from West, East, and Southern Africa ^13^. The absence of longitudinal follow-up to evaluate the impact of *APOL1* status on incident CKD, CKD progression, and survival restrict interpretation of current findings.

The study confirmed known associations with HIV infection, hypertension, and diabetes ^2, 30, 31^, but one third of participants with CKD had none of these risk factors. Potential context-specific risk for kidney disease not accounted for in this study include endemic malaria, endemic genitourinary schistosomiasis, genitourinary tuberculosis, ingestion of traditional and over-the-counter medicines, and environmental exposures such as agricultural pesticides and heavy metal toxins ^32– 34^. Such exposures might result in repeated bouts of acute, or acute-on-chronic kidney injury, or comprise the “second hit” needed for *APOL1*-induced kidney injury.

Our findings show that CKD is prevalent and those with HIV infection, hypertension, and diabetes may benefit from screening strategies to control risk and prevent progression. Research is needed to evaluate performance of creatinine-based eGFR equations in African populations and investigate the contribution of genetic and non-traditional risk factors to CKD risk in South Africa.

## Data Availability

WIReDSpace: Dataset from: Chronic kidney disease (CKD) and associated risk in rural South Africa: a population-based cohort study, https://doi.org/10.54223/uniwitwatersrand-10539-33016 ^35^ This project contains the following underlying data: Readme Orginal data in github format plain text data dataset in xlsx Data are available under the terms of the Creative Commons Zero "No rights reserved" data waiver (CC0 1.0 Public domain dedication).
