# Supplementary material for: Chronic kidney disease (CKD) and associated risk in rural South Africa: a population-based cohort study
Source: Wellcome Open Res. 2022 Nov 3;7:236. Originally published 2022 Sep 20. [Version 2] doi: 10.12688/wellcomeopenres.18016.2 (PMC9674890; doi:10.12688/wellcomeopenres.18016.2)
Supplement: Peer review history

# Peer review: Reviewer response for version 2

**DOI:** 10.21956/wellcomeopenres.20536.r53171

**Published:** 2022-11-18

**Authors:** Ifeoma Ulasi, Ugochi Onu

**Affiliations:** 1 Department of Medicine, College of Medicine, University of Nigeria, Enugu, Nigeria; 2 Department of Internal Medicine, Alex Ekwueme Federal University Teaching Hospital, Abakaliki, Nigeria; 3 Department of Medicine, University of Nigeria Teaching Hospital, Enugu, Nigeria

We have reviewed the revised version. We are satisfied.

 Thank you for the opportunity to be part of this review process.

Is the work clearly and accurately presented and does it cite the current literature?

Yes

If applicable, is the statistical analysis and its interpretation appropriate?

Yes

Are all the source data underlying the results available to ensure full reproducibility?

Yes

Is the study design appropriate and is the work technically sound?

Yes

Are the conclusions drawn adequately supported by the results?

Yes

Are sufficient details of methods and analysis provided to allow replication by others?

Yes

Reviewer Expertise:

NA

We confirm that we have read this submission and believe that we have an appropriate level of expertise to confirm that it is of an acceptable scientific standard.

# Peer review: Reviewer response for version 1

**DOI:** 10.21956/wellcomeopenres.19975.r52492

**Published:** 2022-10-19

**Authors:** Ifeoma Ulasi, Ugochi Onu

**Affiliations:** 1 Department of Medicine, University of Nigeria Teaching Hospital, Enugu, Nigeria; 2 Department of Medicine, College of Medicine, University of Nigeria, Enugu, Nigeria; 3 Department of Internal Medicine, Alex Ekwueme Federal University Teaching Hospital, Abakaliki, Nigeria

Thank you for inviting us to review this manuscript. Knowing that there are not many large population community studies on kidney disease emanating from Africa, this is a step in the right direction. Most of the CKD data in Africa are hospital-based.

 The paper presents a much-needed perspective to studying kidney disease in Africa, looking at traditional risk factors as well as chronic viruses (HIV, hepatitis B virus except for hepatitis C virus) and some other non-traditional risk factors. 

 The strengths of this study, including the large number of adults recruited (66,817), are quite commendable, as rural community recruitment of participants for a study is certainly an enormous task. However, the reason for selecting adults from 20 years of age was not stated. Recruiting participants from 18 years recognized globally as the onset of adulthood may have been more inclusive and good for comparison with other studies.

 Furthermore, there is still a paucity of data on genetic studies in Africa, including the link between CKD and genes; this study going beyond the traditional CKD risk factors in Africa to include genomics is remarkable. 

 We agree and applaud the use of CKD-EPI without race/ethnicity adjustment. Still, it will be good to include that with race/ethnicity adjustment for clarity and future reference. The jury is not quite out in considering the use or not of the race/ethnicity adjustments.

 It will be nice to state how many hypertensive participants had pre-existing hypertension or incidental diagnosis. Similarly, prior vs. incidental HIV diagnosis should have been documented.

 At the first screening, certain patients had hypertension and/or impaired renal function (eGFR /albuminuria); any intervention done between the initial and second screenings should have been documented. Perhaps, these interventions could explain the drop in the number of participants with eGFR <60mls/min/1.73m ^2 ^or albuminuria from 220 to 118 and 29 to 12, respectively.

 Though the statistical analysis seems adequate, a statistician may also review that aspect.

Is the work clearly and accurately presented and does it cite the current literature?

Yes

If applicable, is the statistical analysis and its interpretation appropriate?

Yes

Are all the source data underlying the results available to ensure full reproducibility?

Yes

Is the study design appropriate and is the work technically sound?

Yes

Are the conclusions drawn adequately supported by the results?

Yes

Are sufficient details of methods and analysis provided to allow replication by others?

Yes

Reviewer Expertise:

NA

We confirm that we have read this submission and believe that we have an appropriate level of expertise to confirm that it is of an acceptable scientific standard, however we have significant reservations, as outlined above.

# Response

**Published:** 2022-10-26

**Authors:** June Fabian

**Affiliations:** University of Witwatersrand, South Africa

Thank you for your review. 

 Response to reviewers:

 1. "The strengths of this study, including the large number of adults recruited (66,817), are quite commendable, as rural community recruitment of participants for a study is certainly an enormous task."

- *To clarify, the Agincourt Health and Demographic Surveillance System had a population of 66,817 adults aged 20-79 years from which our representative sample of 2021 consented participants was derived (Figures 1 and 2).*

*2. "*We agree and applaud the use of CKD-EPI without race/ethnicity adjustment. Still, it will be good to include that with race/ethnicity adjustment for clarity and future reference. The jury is not quite out in considering the use or not of the race/ethnicity adjustments."

*Thank you for your comments. We would like to bring the following to the reviewers' attention, as this addresses your concerns:*

- *As a sequel to this study, we selected a subgroup of participants stratified by eGFR and sex (N=986) for a measured GFR study using iohexol as the reference. We showed that inclusion of race-based coefficients overestimated GFR in this rural South African population, and our findings were replicated in our partner country study sites, Malawi and Uganda. Please see reference:*

*https://www.thelancet.com/journals/langlo/article/PIIS2214-109X(22)00239-X/fulltext*

*Fabian J, Kalyesubula R, Mkandawire J, et al.: Measurement of kidney function in   Malawi, South Africa, and Uganda: a multicentre cohort study. The Lancet Global Health. 2022;10(8):e1159-e1169.*

*Given the biodiversity of African populations, we cannot assume our findings are generalisable. However, the participants in the iohexol measured GFR study were the same as those in our population-based sample, and  we have shown unequivocally that race-based coefficients overestimate measured GFR in this population, so this effect on eGFR is clear. As such, we do not feel it is necessary to include the eGFR results with a race-based adjustment in this paper, as it has been addressed separately with reference to measured GFR - the gold standard for evaluating performance of eGFR equations.*

*To make this clear in the revised manuscript, we added the above reference to the methods section "Chronic kidney disease prevalence" where we state "eGFR was calculated using the CKD-EPI _(creatinine)_ equation 2009 without adjusting for African American ethnicity as race-based coefficients have been shown to overestimate GFR in this rural South African population". (tracked changes)*

 3. *"*It will be nice to state how many hypertensive participants had pre-existing hypertension or incidental diagnosis. Similarly, prior vs. incidental HIV diagnosis should have been documented."

*Thank you, we collected these data, stratified by sex:*

*Women: HIV prevalence was 23.8% (278/1170). Of those who tested positive: *

- *259/278 (93.2%) had prior knowledge of their status*
- *15/278 (5.4%) had previously tested negative*
- *4/278 (1.4%) had no prior testing*

*Men: HIV prevalence was 12.0% (102/851). Of those who tested positive: *

- *88/102 (86.2%) had prior knowledge of their status*
- *7/102 (6.9%) each, had previously tested negative or had no prior testing*

*Women: Hypertension prevalence was 15.6% (182/1170). Of those with hypertension: *

- *90/182 (49.5%) were previously tested and informed they were hypertensive*
- *78/182 (42.9%) were previously tested and informed they were normotensive *
- *14/182 (7.7%) had not been tested *

*Men: Hypertension prevalence was 22.0% (187/851). Of those with hypertension: *

- *55/187 (29.4%) were previously tested and informed they were hypertensive*
- *93/187 (49.7%) were previously tested and informed they were normotensive *
- *39/187 (20.9%) had not been tested *

*These data for HIV and hypertension have been added to the footnote for Table 1 (tracked changes).*

 4. "At the first screening, certain patients had hypertension and/or impaired renal function (eGFR /albuminuria); any intervention done between the initial and second screenings should have been documented. Perhaps, these interventions could explain the drop in the number of participants with eGFR <60mls/min/1.73m2 or albuminuria from 220 to 118 and 29 to 12, respectively."

*Thank you for raising this point. At baseline screening, participants with hypertension, HIV (not on treatment), anaemia, hypercholesterolaemia, and hyperglycaemia were referred to their local care facility for further investigation and/or treatment – made possible because tests were performed in real time using point of care technology. The eGFR and albuminuria testing was not done in real time. Rather, samples were batched and shipped for laboratory testing to Johannesburg, South Africa. After obtaining results for kidney function from the laboratory, those with low eGFR (<60ml/min/1.73m ^2^) and/or albuminuria (urine albumin:creatinine ratio >3mg/mmol) were rescreened after a minimum of 3 months (KDIGO CKD diagnosis guidelines). There were no interventions during this minimum 3-month period based on kidney function. However, there may have been some overlap between those referred because of their point of care results (who may also have had low eGFR and/or albuminuria). We have addressed this as a limitation in the revised manuscript (tracked changes).*

 5. "Though the statistical analysis seems adequate, a statistician may also review that aspect."

*Thank you. A biostatistician, Dr Petra Gaylard (co-author in the paper) performed the statistical analysis.*

# Peer review: Reviewer response for version 1

**DOI:** 10.21956/wellcomeopenres.19975.r52489

**Published:** 2022-10-07

**Authors:** Jennifer Lees

**Affiliations:** 1 School of Cardiovascular and Metabolic Health, University of Glasgow, Glasgow, UK

I enjoyed reading this study investigating the prevalence of CKD – as defined by current international guidelines – for the diagnosis of chronic kidney disease in a rural south African population. The authors have further explored potential risk factors for CKD relevant to an African population. The authors acknowledge that the risk factors explored are not exhaustive, and they have detailed other potential risk factors that it was not possible to assess in this study.

 The manuscript is clearly written. The methods are thoroughly detailed and appropriate for the current investigation. Sensitivity analyses are appropriate and justified in the text. The results are clearly displayed in tables and described in text. The strengths and limitations of the study are clearly described. I agree with the authors that there is a major limitation in identifying prevalence of (and risk factors for) CKD in African populations using creatinine-based eGFR, when these estimates of kidney function and the guidelines for their use have been primarily derived and validated in developed countries.

 I believe the manuscript is scientifically valid in its current form. I have only a couple of very minor comments for clarity:

**Abstract and throughout:**

- “WHO” is not defined (though presumably is World Health Organisation) – please specify on first use

**Methods, section on “Chronic kidney disease prevalence”:**

- Authors wrote: “as these coefficients overestimate GFR in African populations”. Can the authors provide a reference for this statement?

Is the work clearly and accurately presented and does it cite the current literature?

Yes

If applicable, is the statistical analysis and its interpretation appropriate?

Yes

Are all the source data underlying the results available to ensure full reproducibility?

Yes

Is the study design appropriate and is the work technically sound?

Yes

Are the conclusions drawn adequately supported by the results?

Yes

Are sufficient details of methods and analysis provided to allow replication by others?

Yes

Reviewer Expertise:

Estimated GFR equations; CKD epidemiology (risk factors and outcomes)

I confirm that I have read this submission and believe that I have an appropriate level of expertise to confirm that it is of an acceptable scientific standard.

# Response

**Published:** 2022-10-26

**Authors:** June Fabian

**Affiliations:** University of Witwatersrand, South Africa

Thank you for your review. 

 As requested, I have included the full name for the "World Health Organization" (WHO) on first use; and I have added a reference for the statement "as these coefficients overestimate GFR in African populations" in the methods for the section titled "Chronic kidney disease prevalence"

 The above changes are tracked in the revised version of the paper that has been resubmitted.
